# Assessment of Circulating miRNA-17 and miRNA-222 Expression Profiles as Non-Invasive Biomarkers in Egyptian Patients with Non-Small-Cell Lung Cancer

**DOI:** 10.31557/APJCP.2019.20.6.1927

**Published:** 2019

**Authors:** Helal F Hetta, Asmaa M Zahran, Reham I El-Mahdy, Emad Eldin Nabil, Hend M Esmaeel, Ola A Alkady, Azza Elkady, Dina A Mohareb, Mohammed Mahmoud Mostafa, James John

**Affiliations:** 1 *Department of Internal Medicine, University of Cincinnati College of Medicine, Cincinnati, OH, USA, *; 2 *Department of Medical Microbiology and Immunology, *; 4 *Department of Medical Biochemistry, *; 8 *Department of Clinical Pathology, Faculty of Medicine, *; 9 *Department of Cardiothoracic Surgery, Assiut University Hospital, Assiut University, *; 3 *Department of Clinical Pathology, South Egypt Cancer Institute, Assiut, *; 5 *Department of clinical Oncology, *; 6 *Department of Chest Diseases and Tuberculosis, Faculty of Medicine, Sohag University, *; 7 *Sohag general hospital, Sohag, Egypt, *; 10 *Central Research Facility, Sri Ramachandra Medical College and Research Institute, Sri Ramachandra University, Chennai, India. *

**Keywords:** Biomarker, miR-17, miR-222, non-invasive, non-small-cell lung cancer

## Abstract

**Background:**

Lung cancer is one of the main human health threats. Survival of lung cancer patients depends on the timely detection and diagnosis. Among the genetic irregularities that control cancer development and progression, there are microRNAs (miRNAs). This study aimed to assess the plasma level of circulating miRNA-17 and miRNA-222 as non-invasive markers in non-small-cell lung cancer (NSCLC) patients.

**Patients and methods:**

A total of 40 patients with NSCLC and 20 healthy controls who were matched in terms of age and sex with the patient group were included in this case-control study.. Estimation of miRNA-17 and miRNA-222 expression profiles in the plasma was done using quantitative real-time PCR (qRT-PCR). The relationship between both markers and their clinicopathological features were also determined. Receiver operating characteristic (ROC) curve analysis was done to evaluate the role of these microRNAs in NSCLC diagnosis and follow-up.

**Results:**

MiRNA-17 and miRNA-222 levels were significantly upregulated in NSCLC patients compared with controls (48.32±12.35 vs 1.16±0.19 and 34.53±3.1 vs 1.22±0.14) (P=0.000). Plasma miRNA-17 level was increased, and the miRNA-222 level was decreased across different stages of the disease; however, these differences d were not statistically significant (P=0.4, P=0.5, respectively). The miRNA-17 levels were higher in the lung cancer patients with metastasis , but miRNA-222 levels were lower patients without metastasis. We found no statistically significant difference in this regard(P=0.4 vs P=0.3, respectively). ROC curve analysis showed that the sensi¬tivity and specificity of miRNA-17 were 77.78% and 87.50% , and of miRNA-222 were 50% and 88.89%.

**Conclusion:**

MiRNA-17 and miRNA-222 can be considered as non-invasive biomarkers for detection of early lung carcinogenesis and metastasis in patients with NSCLC, hence providing a basis for the development of novel therapeutic approaches.

## Introduction

Lung cancer is one of the most important leading causes of cancer death in both men and women in developed as well as developing countries. Every year more than 1.5 million deaths caused by lung cancer are reported (Wang et al., 2018). Non-small-cell lung cancer (NSCLC) is the most commonly detected type of lung cancer. Despite advances in cancer management, NSCLC prognosis is still poor, given that the 5-year survival rate of patients is less than 18% which is partly due to the fact that most of patients with NSCLC are diagnosed at late stages (Siegel et al., 2016). An emerging approach for NSCLC diagnosis and therapeutics is based on microRNAs (Inamura and Ishikawa, 2016).

MicroRNAs are the novel class of endogenously expressed, single-stranded, short non-coding RNAs of 18–22 nucleotides in length. MicroRNAs play controlling roles over the posttranscriptional expression of the majority of the human protein-coding genes (Melo and Esteller, 2014). They are involved in many physiological processes, including cell differentiation, growth, proliferation, apoptosis, motility, and survival (Lucas and Raikhel, 2013). The frequent dysregulated expression patterns of miRNAs in cancers have been identified, which are reflected in the circulation (Li et al., 2017). Circulatory miRNAs have shown a distinguished constancy in body fluids (Mitchell et al., 2008). Thus, miRNAs are regarded as non-invasive biomarkers for diagnosis, prognosis, and treatment of NSCLC (Peng and Croce, 2016).

MiRNAs transcribed from the nearby miRNA genes are called cluster. MiRNA-17 is a member of the miRNA-17 family that includes miRNA-17-18-19a-20-19b-92 cluster. It is situated on a human chromosome 13q31.3 (Tagawa et al., 2007). Recent studies have confirmed the close relationship between the expression level of miRNA-17 and several types of human cancers, but the precise role of miRNA-17 in cancer remains controversial. Some scientists have suggested that miRNA-17 may act as a tumor suppressor, but other studies have suggested it as an oncogene (Zhang et al., 2018).

MiRNA-222 is a member of the miRNA-221/222 family, which plays a critical role in multiple cancers (Liu et al., 2014). Down-regulation of the miRNA-221/222 is associated with poor patient outcomes and chemoresistance in NSCLC (Sathipati and Ho, 2017). The miR-221/222 sequence is highly conserved and commonly binds to small regions at its targeting gene 5′ ends. A large number of research indicates that these two miRNAs frequently mark numerous high expression genes in epithelial malignancies, such as glioma, prostate carcinoma, hepatocellular cancer, and breast cancer (Wei et al., 2014).

Accordingly, this study aimed to assess the plasma expression patterns of miRNA-17 and miRNA-222 in NSCLC patients and examine the possibility of using miRNA-17 and miRNA-222 as non-invasive biomarkers for NSCLC.

## Materials and Methods

*Patients and methods*

The current case-control study was performed on 40 NSCLC patients (19 smokers and 21 non-or ex-smokers) and 20 controls. The patients were selected randomly from those who referred to Chest, Cardiothoracic Surgery, and Clinical Oncology Departments, Sohag and Assiut University hospitals, Egypt, between December 2017 and May 2018. The patients included 23 males and 17 females. The mean age of the patients was 59.95±9.74 years old. The controls were matched with the case group in terms of age and sex. There were 40 NSCLC patients in the case group and 20 healthy individuals in the control group. The case group included 19 patients with adenocarcinoma, 14 with squamous cell carcinoma, 4 with undifferentiated carcinoma, and 3 with large cell carcinoma. 

All patients underwent bronchoscopy (Pentax EB 1970TK) and/or image-guided tru-cut needle biopsy from lung mass lesion, histopathological examination of lung mass biopsy, computed tomography chest study to evaluate the primary, computed tomography study for the brain, neck, abdomen and pelvis to assess the presence or absence of distant visceral metastasis, nuclear bone scanning to detect the presence of bone metastasis, complete blood picture, complete liver function and kidney function tests. Clinical staging was done using previous tools and TNM staging system. None of the patients were exposed to radical surgery for tumor resection or lymph node dissection; hence, no pathological staging was available. Samples were collected from patients before starting any anti-cancer treatments.

Patients with pulmonary infections or TB, chronic medical disease (cardiac, hepatic, or renal), and other malignancies as well as patients who received neoadjuvant chemotherapy for lung cancer prior to the study were excluded. All participants in both groups signed written informed consents for participating in the study. The study was conducted in accordance with the Declaration of Helsinki and approved by the local Clinical Research Ethics Committee.

*Plasma specimen collection*

Five milliliters of venous blood were collected from each participant, kept in an EDTA-anticoagulant tube (BD, Franklin Lakes, NJ, USA), and centrifuged at 2,000 rpm for 10 minutes. Plasma was transmitted to a fresh tube and preserved at –80°C until use.

*RNA extraction and reverse transcription*

Total RNA, including miRNA, was extracted from plasma using a Direct-zol™ RNA MiniPrep Kit (Zymoresearch, Catalog No. R2053, CA, USA). The RNA purity and concentration were detected using a Biotech Nanodrop system. Poly (A) polymerase enzyme (NEB, New England; Cat.no. M0276L) was used to increase the poly A tail of small noncoding miRNAs, and reverse transcription was done with a Thermo Scientific Revert Aid Reverse kit (Thermo, Waltham, MA, US). In addition, cDNA was collected after transcription.

*QRT-PCR analysis of miRNAs*

Real-time quantitative PCR was performed to assess the expression level of miR17 and miR222 using Hs_miR-17_2 miScript Primer Assay (Cat number MS00029274, Qiagen, USA) and Hs_miR-222_2 miScript Primer Assay (Cat number MS00007609, Qiagen, USA) respectively.

Using MiScript SYBR Green PCR kit (Cat number 218073, Qiagen, USA), the reaction was carried out on 7500 Fast Real-Time PCR System (Applied Biosystems, CA, USA) as follows: a hot-start step at 95°C for 7 min, followed by an initial denaturation for 20 sec at 95ºC and annealing and extension for 60 sec at 59 ºC for 40 cycles.

The relative miRNA-17 and miRNA-222 expression levels were calculated using the following equation: fold change= 2^−ΔΔCT^. RNU6-2 (Hs_RNU6-2_11 miScript Primer Assay, MS00033740) was used as endogenous control for the normalization of the expression levels of miRNA-17 and miRNA-222.

*Statistical analysis*

Data were analyzed by SPSS (version 20) and Graph Pad Prism 6 Software (San Diego, California, USA). The data were presented as mean, standard deviation (SD), standard error means (SEM), and percentage. The cut-off point, sensitivity, specificity, and the area under the ROC curve were obtained using MedCalc program. Prior to analysis, the variables were tested for normal distribution using the Shapiro-Wilk W test. Student t-test was used for normally distributed data and Mann-Whitney was used for skewed data to identify possible differences between groups. ANOVA test was used for comparison of groups. Spearman correlation was used to detect any correlations between various parameters. Differences were considered significant at p≤0.05. 

## Results

*NSCLC patient’s demographic and clinical data*

The demographic data and the clinicopathological characteristics of NSCLC patients are presented in Table 1. Most of the patients were at stage IV (50%) and stage III (37.5%), respectively . In addition, most of the histopathological types of cancer were adenocarcinoma (47.5%).

According to Table 2, there was a significant difference between two groups concerning the expression levels of plasma miRNA-17 (48.32±12.35 vs 1.16±0.19 fold change) and miRNA-222 (34.53±3.1 vs 1.22±0.14 fold change) (P=0.000).

No significant difference in the expression pattern of miRNA-17 and miRNA-222 was detected between the smokers (n=19) and non-smokers (n=21) (P=0.782 and P=0.700, respectively), and between patients aged >50 years old (n=30) and those aged <50 years old (n=10) (P=0.754 and P=0.340, respectively) (data not shown). 

*Expression pattern of miRNA-17 and miRNA 222 levels in the NSCLC patients regarding different TNM classifications*

**Figure 1:**
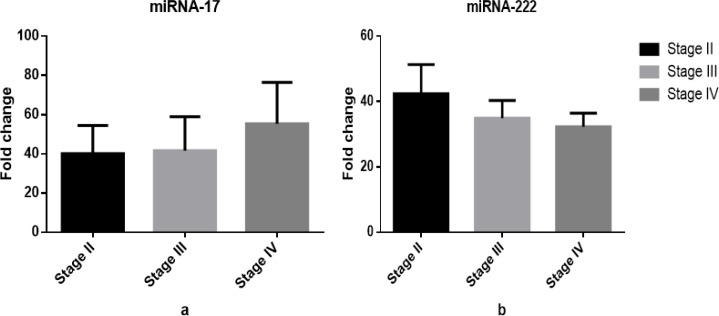
MiRNA-17and mi-RNA 222 Expression Patterns in NSCLC Patients (n=40) Regarding Different Malignant Stages of Lung Cancer. (a) Plasma level of mi-RNA 17 in NSCLC patients with different malignant stages (P=0.4) , (stage II 40.09±14.36, stage III 41.62±17.3, stage IV 55.41±21.08). (b) Plasma level of mi-RNA 222 in NSCLC patients with different stages (P=0.5), (stage II 42.42±8.9, stage III 34.93±5.4, stage IV 32.25±4.2)

Plasma miRNA-17 level was increased (stage II 40.09±14.36, stage III 41.62±17.3, stage IV 55.41±21.08), but the miRNA-222 level was decreased (stage II 42.42±8.9, stage III 34.93±5.4, stage IV 32.25±4.2) across different stages. However, this different was not statistically significant (P=0.4 vs. P=0.5)(Figure 1).

**Figure (2):**
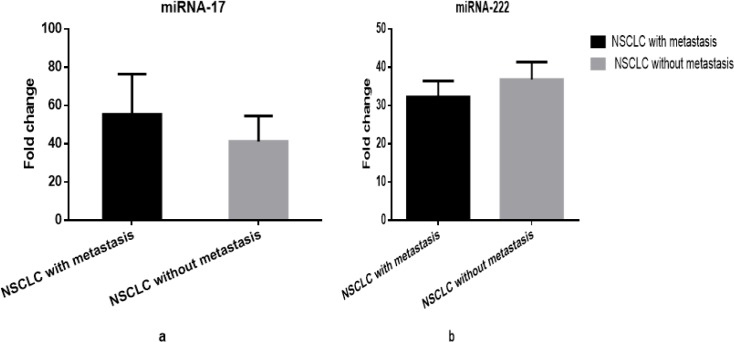
The Expression Fold Change of Circulating mi-RNA 17 and mi-RNA 222 Using qRT-PCR in NSCLC Patients with Different Clinical Status. (a) Plasma level of mi-RNA 17 in NSCLC patients with distant metastasis (55.41±21.08) versus patients without metastasis (41.24±13.28) (P=0.4). (b) Plasma level of mi-RNA 222 in NSCLC patients with metastasis (32.25±4.2) versus patients without metastasis (36.80±4.6) (P=0.3). P values were calculated using student t-test. Data are presented as “Mean±SEM fold change”

As it is clear from Figure 2, there was an increase in the plasma level of miRNA-17 and decrease in miRNA-222 expression profile in the NSCLC patients at different clinical phases (with distant metastasis (P=0.445) vs. without distant metastasis (P=0.358)), but it did not lead to statistically significant this difference.

*Expression profile of miRNA-17 and miRNA-222 in different histological types*

**Figure 3:**
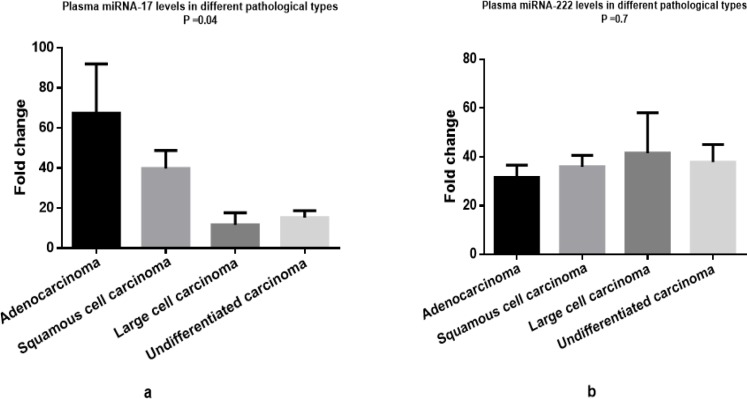
Mi-RNA 17 and mi-RNA 222 Expression Levels in NSCLC Patients Regarding Different Histopathological Types. A significant difference in plasma level of mi-RNA 17 in NSCLC patients with different histopathological types (P =0.04*) was found while there was no significant difference in plasma level of mi-RNA 222 in NSCLC patients with different histopathological types (P =0.7).*, P<0.05 is considered significant. Data are presented as “Mean±SEM fold change”

There was a significant difference among 4 histological types in terms of plasma miRNA-17 level (P=0.04*) as shown in Figure 3 (the peak values were found in adenocarcinoma 67.39±24.57, followed by squamous cell carcinoma 39.77±9.00, undifferentiated carcinoma 15.28±3.40, and large cell carcinoma 11.64±6.10). Nevertheless, the results displayed no significant differences among these 4 types considering plasma miRNA-222 level (31.66 ±4.95, 35.95±4.66, 37.88±7.23, and 41.57±16.51 respectively) (P=0.7), as revealed in Figure 3.

*The accuracy of the miRNA-17 and miRNA-222 levels for NSCLC diagnosis and discrimination using ROC curves*

**Figure 4:**
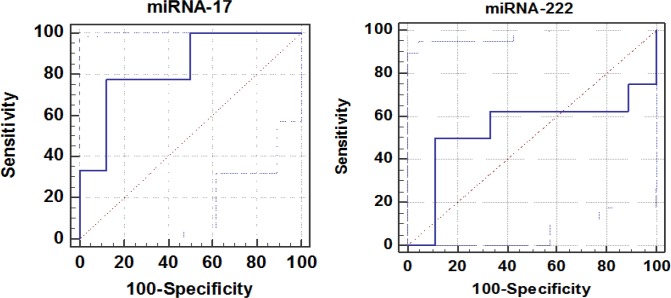
Receiver Operating Characteristic (ROC) Curve for miRNA-17 and miRNA-222 ROC Curve for miRNA-17: The area under the ROC was 0.833, (Standard error was 0.105, 95% confidence interval was 0.577 to 0.966). The specificity, sensitivity, and cut-off point were at 87.50%, 77.78%, and 29.899 fold change, respectively. ROC curve for miRNA-222: The area under the ROC was 0.542, (Standard error was 0.167, 95% confidence interval was 0.289 to 0.780), the specificity, sensitivity, and cut-off point were at 88.89%, 50.00%, and 12.927 fold change , respectively

The sensitivity, specificity, and area under the curve for plasma miRNA-17 and miRNA 222 in NSCLC patients are shown in Figure 4 based on the cut-off levels. Plasma miRNA-17 showed greater area under the ROC curve (0.833) with 77.78% sensitivity and 87.50% specificity. This finding indi¬cated that the miRNA-17 level may be a valuable index for discriminating NSCLC patients from healthy controls.

**Table 1:** The Demographic and the Clinicopathological Characteristics of the Non-Small Cell Lung Cancer Patients

Variables	Non-Small Cell Lung Cancer patients (n=40)
Age (years)	
mean ± SD	59.95±9.74
Sex n (%)	
Male	23 (57.5%)
Female	17 (42.5%)
Smoking habits	
Non or ex-smoker, n (%)	21 (52.5%)
Current smoker, n (%)	19 (47.5%)
Histopathological type, n (%)	
Adenocarcinoma	19 (47.5%)
Squamous cell carcinoma	14 (35%)
Large cell carcinoma	3 (7.5%)
Undifferentiated carcinoma	4 (10%)
Chemotherapy cycles number (mean ± SD)	3.07±0.86
Grade	
Grade II	17 (42.5%)
Grade III	17 (42.5%)
Grade IV	6 (15%)
TNM classification, n (%)	
Tumor size (T)	
T2	8 (20%)
T3	26 (65%)
T4	6 (15%)
Intrathoracic Lymph node involvement (N)	
N0	8 (20%)
N1	10 (25%)
N2	19 (47.5%)
N3	3 (7.5)
Distant metastasis (M)	
M0	20 (50%)
M1	20 (50%)
Malignant stage	
Stage II	5 (12.5%)
Stage III	15 (37.5%)
Stage IV	20 (50%)

TNM, tumor-nodal-metastasis classification; M0, no distant metastasis; M1, distant metastasis

**Table 2:** Expression Levels of mi-RNA 17 and mi-RNA 222 in NSCLC Patients Versus Healthy Controls

Variables	Patients (n=40)	Controls (n=20)	p value
	Mean±SEM	Mean±SEM	
miRNA 17	48.32±12.35	1.16±0.19	0
miRNA 222	34.53±3.1	1.22±0.14	0

P values were calculated using ANOVA test. Data are presented as “Mean±SEM fold change”

## Discussion

Lung cancer ranks first as a cause of cancer death worldwide, which is categorized with late stage diagnosis and an unfavorable outcome (Liu et al., 2018). In clinical practice, NSCLC is the most common type of lung carcinoma. It represents more than 80% of all cases and sub-classified into adenocarcinomas, squamous cell, large cell, and undifferentiated carcinomas (Yang et al., 2018). NSCLC can be cured with surgical procedure alone or with adjuvant chemotherapy; although, approximately half of the patients experience worse survival, disease recurrence, and death because of resistance to drug. Therefore, there is a crucial need to find novel precise non-invasive tools for early diagnosis, predictive stratification, and effective treatment of patients (Zhang et al., 2018).

MicroRNAs, as short non-coding RNA molecules, have been found to regulate gene expression post-transcriptionally by repressing the translation or inducing messenger RNA degradation, hence playing critical roles in carcinogenesis through various mechanisms. Certain miRNAs have been identified to be interrelated with patients’ clinical characteristics and outcomes (Chen et al., 2012). Plasma or serum miRNA can originate from lung cancer cells and reflect disease activity due to the high blood perfusion of the tumors (Mitchell et al., 2008).

MiRNA-17, also known as miR-17-5p or OncomiR-1, is the main active product of polycistronic miR-17-92 cluster (Wang et al., 2018). A possible mechanism by which miRNA-17 promotes cancer onset and progression is enhancement of cell proliferation through modulation of the PI3K/Akt/mTOR pathway. Moreover, miR 17 92 prevents tumor suppressor genes phosphatase and tensin homolog by triggering the protein kinase B signaling pathway to induce cancer cell survival (Tan et al., 2018). MiR-221 and miR-222 carry the same sequence. This sequence is evolutionarily conserved and often binds to short areas at its targeting gene 5ˊ ends (Wang et al., 2018). MiR-222 can enhance cellular migration through activation of AKT pathway and metallopeptidases in NSCLC (Xu et al., 2018).

In the present study, assessment of the expression patterns of the miRNA-17 and miRNA-222 in NSCLC and their association with clinicopathological indices was done. Our data showed that circulating plasma miRNA-17 and miRNA-222 expression levels were significantly higher in patients with NSCLC than those in healthy controls (P=0.000). In accordance with our findings, previous studies have shown that aberrant miRNA expression in NSCLC patients’ blood and healthy individuals is powerfully associated with cancer development and progress (Xu et al., 2015; Zaporozhchenko et al., 2018; Yang et al., 2018; Liu et al., 2018; Liu and Wang, 2017). Contrary to such speculations, Li et al., (2013) have revealed that the expression of miR-17-5p is reduced in triple-negative breast cancer tissues and that it can suppress tumor progression through inhibition of ETV1 oncogene in triple-negative breast cancer cells. This is due to dysregulated expression of miRNA in various cancers.

MiRNA-17 has a key oncogenic function in cancers (Wang et al., 2018). Numerous studies have described the overexpression of miR-17 for another type of cancers, such as brain cancer (Gruszka and Zakrzewska, 2018), hepatocellular (Giray et al., 2014), and colon (Elshafei et al., 2017). The plasma expression level of miRNA-17 was raised at advanced NSCLC stages (Figure 1), but it did not lead to a statistically significant difference between two groups. These results are in a line with those reported by Hayashita et al., (2005), Latchana et al., (2017), and Yin et al., (2017), demonstrating an association between the up-regulation of plasma miR-17 expression and advanced clinical stage as well as aggressive form of cancer. Concerning miRNA-222, the present study found that its expression level in NSCLC patients was reduced at late stages (Figure 1). Additionally, plasma miRNA-222-fold change levels were lower in patients with metastasis versus those without metastasis (Figure 2). Similarly, Goto et al., (2015) have indicated that lower miRNA-222 expression level is associated with aggressive prostate cancer stages and metastasis potentials. 

Interestingly, our data revealed that miRNA-17 levels in the lung cancer patients with metastasis were higher than those in the patients without metastasis (Figure 2), as secondaries may produce miRNA-17. However, it was a non-statistically significant difference between two groups (P=0.4). In similar vein, Fu et al., (2018) found that miRNA-17 expression level was related to metastasis in lymph nodes. The cellular microenvironment is important for the start and progress of tumorigenesis. Similar to primary carcinogenesis, metastasis of malignant growth needs particular microenvironments in distant metastatic tissues which enables the assembly, colonization, and propagation of flowing tumor cells. Cancer cell-derived exosomes with prometastatic influences, including miRNAs, have been found to induce and aid premetastatic niche creation, allowing the survival and extension of dispersed tumor cells (Fu et al., 2018). Furthermore, the results of the present study found no significant difference between younger and older as well as smokers and non-smokers patients in terms of plasma miRNA-17 and miRNA-222 expression profile. These results are consistent with the findings reported by Liu et al., (2013) and Gruszka and Zakrzewska (2018). 

To analyze the diagnostic probability of miRNA-17 and miRNA-222, the ROC curve was made, yielding that miRNA-17 had the best cutoff point in distinguishing NSCLC from controls with 77.78% sensitivity, 87.50% specificity, and greater area under the ROC curve (0.833) (Figure 4). This finding is in accordance with the results of other studies on different types of cancer (Tagawa et al., 2007; Chang et al., 2016; Gruszka and Zakrzewska, 2018). Another study also has displayed a hopeful diagnostic and prognostic value of miRNA-17 in discriminating NSCLC patients from healthy controls and detecting the tumor recurrence earlier than the appearance of symptoms and abnormal CT images (Wang et al., 2018). 

Concerning the histopathologic types of NSCLC, the present study found that plasma miRNA-17 level was significantly upregulated in NSCLC patients with adenocarcinoma compared with other histological types (P=0.04). However, we no significant difference concerning plasma miRNA-222 level among these 4 types (P=0.7). Adenocarcinoma is the commonest noticed type of NSCLC (Yang et al., 2018). Targeting microRNA17 could control the expression of downstream intermediates of cancer development and progression. Consequently, therapeutic approaches to decrease microRNA17 may be valuable to prevent NSCLC development and metastasis. Additional effort is necessary to assess the role of microRNA17 and identify the downstream targets *in vivo* (Wang et al., 2018). 

We faced with some inevitable limitations in the current investigation. on the first limitation was the small size of the statistical population. Therefore, future studies are recommended to be done on larger sample size in order to approve or disapprove these results. The second limitation was lack of NSCLC patients follow up to assess the plasma miRNA levels after different therapies, which could not be done due to death of the patients, their far distant from the hospital, or their deprived socioeconomic status.

In conclusion, our study revealed that miRNA-17 and miRNA-222 can be considered non-invasive sensitive and specific biomarkers for NSCLC diagnosis and therapeutic response prediction.
